# Factors Influencing Post-Traumatic Stress Disorder in Hospital Clinical Nurses during COVID-19 in Korea: Resilience, Social Support, and Professional Pride in Nursing

**DOI:** 10.3390/healthcare12141401

**Published:** 2024-07-14

**Authors:** Bomi Kim, Hae Ran Kim, Jae Yong Yoo, Mi Ah Han

**Affiliations:** 1 Department of Nursing, Chosun University Hospital, Gwangju 61453, Republic of Korea; kbomee@naver.com; 2 Department of Nursing, Chosun University College of Medicine, Gwangju 61452, Republic of Korea; jaeyongyoo@chosun.ac.kr; 3 Department of Preventive Medicine, Chosun University College of Medicine, Gwangju 61452, Republic of Korea; mahan@chosun.ac.kr

**Keywords:** COVID-19, post-traumatic stress disorder, resilience, social support, professional pride

## Abstract

During the COVID-19 pandemic, clinical nurses in hospitals in South Korea were exposed to extreme stress, and many continue to suffer from post-traumatic stress disorder (PTSD). This study explores the factors influencing PTSD among hospital clinical nurses during COVID-19. In total, 121 hospital clinical nurses participated in 2022, providing demographic information and completing surveys designed to measure PTSD, resilience, social support, professional pride in nursing (PPN), and variables related to COVID-19. We observed statistically significantly higher levels of resilience (91.48 vs. 70.00), social support (47.37 vs. 35.41), and PPN (88.36 vs. 68.06) in the low-risk PTSD group compared with the high-risk PTSD group. Resilience was associated with a reduced risk of PTSD (OR, 0.91; 95% CI = 0.84–0.98). The subfactors of control (OR = 0.60; 95% CI = 0.43–0.86) and sociability (OR = 0.68; 95% CI = 0.44–0.97) decreased PTSD risk. Among the social support subfactors, family support had an OR of 0.47 (95% CI = 0.26–0.86) for reducing PTSD risk. Programs involving family participation that enhance resilience and provide psychological support can help hospital nurses affected by the COVID-19 pandemic manage their PTSD. Our findings serve as foundational data to develop interventions on psychological well-being for nurses dealing with new infectious diseases.

## 1. Introduction

Korea was severely hit by COVID-19. The rapid spread of the virus led to widespread infections domestically, causing significant disruptions and hardships in both workplaces and daily life. Despite rigorous government efforts to contain the spread through strong public health measures, the pandemic posed substantial challenges in the healthcare sector and brought about various socio-economic issues [1]. During the 2019 coronavirus pandemic (COVID-19) outbreak, hospital clinical nurses were deployed on the front lines to establish response systems for managing new infectious diseases and provide direct care to patients infected with COVID-19. Nurses reported experiencing severe anxiety and depression due to the burden of infection and fear of transmission [2,3]. The uncertainty surrounding COVID-19, the lack of or prolonged use of protective equipment, mental fatigue, and physical exhaustion from tasks beyond nursing duties (e.g., dealing with guardians and explaining things to patients) exacerbated nurses’ psychological stress [4].

It is well documented that nurses have experienced post-traumatic stress disorder (PTSD) due to the psychological impact of COVID-19 [5]. PTSD after COVID-19 is characterized by re-experiencing similar events and avoiding situations that trigger memories of trauma, negative psychological changes, and heightened reactions [6]. Due to their professional characteristics, nurses have the highest prevalence of PTSD among healthcare workers [7]. During a pandemic, nurses often focus most of their energy and resources on alleviating patients’ physical illnesses, neglecting their own psychological well-being [8]. Nurses assigned to COVID-19 care departments are closely connected with patients and face physical and mental burnout [4]. PTSD can lead to increased turnover intention, reduced concentration and cognition, medication-related errors, and performance issues that affect patient safety and healthcare institutions [9]. Recent studies in South Korea indicated that approximately 8.9% of nurses report high-risk PTSD [10]. This is significantly higher than the lifetime prevalence of PTSD in the general South Korean population, which was reported to be 1.5% in the 2021 Mental Health Survey by the Ministry of Health and Welfare (MOHW) [11]. Strategies to mitigate PTSD are essential for minimizing its negative impacts, improving nurses’ mental health, and enhancing the quality of healthcare. 

Identifying the factors that influence PTSD is crucial for reducing incidence rates and establishing prevention strategies among nurses. A systematic review of PTSD among healthcare workers suggested that various environmental factors contribute to PTSD [12]. Organizational factors—such as poor working conditions, additional tasks due to infection control, and a lack of physical support—are linked to job stress, the quality of patient care, and turnover intention. Social support is a critical interpersonal resource within social networks that helps improve individual mental health [13]. Low social support can exacerbate the impact of job stress, potentially linking it to PTSD [14]. Resilience, a personal factor characterized by a positive perception of adversity and proactive problem-solving, can serve as a protective factor against PTSD in healthcare workers [15]. A 2022 study in South Korea found that professional pride in nursing (PPN, which fosters confidence and positive emotions about one’s work) helps improve job performance and organizational cohesion [16,17]. This implies that PPN was beneficial in helping nurses provide professional care during the early period of the pandemic. Previous studies on frontline nurses’ experience during COVID-19 have indicated that the incidence of PTSD in nurses is related to their experience with COVID-19, their duration of work experience, and information related to infectious diseases [12,18]. As key frontline healthcare workers in potential post-COVID-19 disaster scenarios, nurses are clearly at risk of adverse mental health outcomes, such as PTSD. It is imperative to address PTSD and enhance nurses’ mental well-being to develop strategies for providing high-quality patient care. Identifying the factors that influence PTSD is essential to identifying at-risk nurses. This study aimed to explore the factors influencing PTSD among nurses who provided care during the COVID-19 pandemic by focusing on resilience, social support, and PPN.

## 2. Materials and Methods

### 2.1. Study Design and Participants

Using a descriptive survey, we aimed to identify the factors influencing PTSD in nurses during the COVID-19 pandemic. This study was approved by the Institutional Review Board of Chosun University Hospital (No.: CHOSUN 2022-06-029-003). In August 2022, we conducted convenience sampling targeting nurses from three comprehensive hospitals in Gwangju, Korea, each with over 300 beds. First, after obtaining approval from the hospital administrators, the researcher explained the study’s purpose and procedures to the nursing departments. Second, questionnaires were distributed to nurses who agreed to participate, facilitated by the nursing departments. The consent form included details about the study’s purpose, voluntary participation, withdrawal and termination rights, and the data-retention period. Third, the survey was self-reported and sealed in an envelope collected by the researcher upon completion. Filling out the survey took approximately 15 min, and the participants’ data remained anonymous. Gift cards were provided to the nurses who completed the survey.

The sample size was determined using the G*Power 3.1.9 program to perform binary logistic regression analysis, yielding a minimum sample size of 115 (two-tailed test, significance level α = 0.05, odds ratio [OR] = 2.1, control group probability [H0] = 0.5, power = 0.95). Considering a 20% dropout rate, 140 questionnaires were distributed, and after excluding incomplete data, 121 responses (86.4%) were analyzed.

### 2.2. Instrumentation

The general characteristics of the participants included gender (male, female), age (20–29 years, 30–39 years, 40 years and above), education level (associate’s degree, bachelor’s degree, master’s degree or higher), and the type of work department (COVID-19 dedicated ward, general ward, emergency room, or intensive care unit [ICU]). The participants’ work schedules were categorized as either day shifts or three-shift rotations. Their positions were classified as staff nurses, charge nurses, or higher. Clinical experience was grouped as less than 5 years, 5–10 years, 11–15 years, and 15 years and above. Additionally, information on whether participants lived with family or others (yes or no) and had chronic diseases (yes or no) was collected to obtain a comprehensive understanding of their backgrounds.

According to previous studies [12,14,18], COVID-19 related variables include the experience of being diagnosed with COVID-19 during the pandemic (yes, no); the experience of being assigned to a dedicated department to care for COVID-19 patients (yes, no); the average number of patients under care during the pandemic (less than 10, 10 or more); the duration of care for confirmed or quarantined COVID-19 patients (less than 12 months, 12 months or more); changes in workload during the pandemic (increase, no change, or decrease); the supply of personal protective equipment (sufficient, insufficient, or unsure); the experience of training in infection patient management (yes or no); the presence of hospital guidelines for managing infectious diseases (yes or no); and the intention to care for patients with new infectious diseases (yes, no, or unsure). 

PTSD was assessed using the Impact of Event Scale-Revised (IES-R-K), which has been validated for reliability and validity in measuring the risk of developing PTSD among nurses in South Korea [19]. This tool comprises the following four subdimensions: hyperarousal (six items); avoidance (six items); intrusion (five items); sleep disturbance; and numbness (five items). The participants responded on a scale ranging from 0 (not at all) to 4 (extremely) based on their experiences over the past month. The score range is 0–88. Scores of 25 or above were defined as a risk of developing PTSD, whereas scores of 24 or below were considered normal. In this study, Cronbach’s α was 0.96.

Resilience was measured using a tool developed by Shin et al. [20]. This tool includes 27 items across 3 subdimensions, i.e., controllability (nine items); positivity (nine items); and sociality (nine items). The participants responded on a scale of 1 (not at all true) to 5 (completely true). The scores ranged from 27 to 135, with higher scores suggesting greater resilience. In this study, Cronbach’s α was 0.91. 

Social support was assessed using the Korean version of the Multidimensional Scale of Perceived Social Support (MSPSS) [21]. This tool consists of 12 items across 3 subdimensions, i.e., family members (four items); friends (four items); and significant others (four items). The participants responded on a scale ranging from 1 (strongly disagree) to 7 (strongly agree). The scores ranged from 12 to 84, with higher scores suggesting greater social support. In this study, Cronbach’s α was 0.95.

PPN was measured using the tool developed by Jeon et al. for Korean nurses [22]. This tool comprises 27 items across 5 subdimensions, i.e., a feeling of vocation (six items); role satisfaction (six items); role of the problem-solver (six items); self-achievement (four items); and willingness to stay (five items). The participants responded on a scale of 1 (not at all true) to 5 (completely true). The scores ranged from 27 to 135, with higher scores implying greater PPN. In this study, Cronbach’s α was 0.90.

### 2.3. Statistical Data Analysis

We performed all statistical analyses using SPSS Version 27 (IBM Corp., Armonk, NY, USA). We calculated the means, standard deviations, frequencies, and percentages to analyze PTSD. We employed the χ^2^ test and an independent *t*-test to analyze PTSD in relation to general characteristics, COVID-19-related variables, resilience, social support, and PPN. We further examined variables that were statistically significant in the univariate analysis using binary logistic regression to identify factors influencing PTSD. We assessed the model’s goodness of fit using the Hosmer–Lemeshow goodness of fit test.

## 3. Results

### 3.1. PTSD Reported by Nurses

The average PTSD score was 16.21 ± 14.87. Among the participants, 28.1% were classified as being at risk for PTSD, with an average PTSD score of 36.15 ± 10.60 (Table 1).

### 3.2. Risk of PTSD by General Characteristics

During the COVID-19 pandemic, 97.06% of nurses in the at-risk-of-PTSD group had experienced a COVID-19 diagnosis (χ^2^ = 6.85, *p* = 0.009), and 44.12% had been assigned to dedicated departments to care for COVID-19 patients (χ^2^ = 4.67, *p* = 0.031) (Table 2).

### 3.3. PTSD Risk: Resilience, Social Support, and PPN

Resilience scores were significantly lower in the at-risk-of-PTSD group than in the control group (91.48 vs. 70.00, *t* = 8.14, *p* < 0.001). The scores for the subfactors of resilience were also lower in the at-risk-of-PTSD group. Social support scores were lower in the at-risk-of-PTSD group than in the normal group (47.37 vs. 35.41, *t* = 7.21, *p* < 0.001), as were the subfactor scores. PPN was also lower in the at-risk-of-PTSD group than in the control group (88.36 vs. 68.06, *t* = 6.97, *p* < 0.001). This trend was consistent across subfactors (Table 3).

### 3.4. Factors Influencing Nurses’ PTSD

To identify the factors influencing nurses’ PTSD, a binary logistic regression analysis was conducted using PTSD risk (normal vs. high at-risk group) as the dependent variable and significant variables from the univariate analysis (COVID-19 diagnosis, reassignment to care for COVID-19 patients, resilience, social support, and PPN) as the independent variables. A significant model (Model I) was derived (χ^2^ = 63.22, *p* < 0.001) with an explanatory power of 59%. Resilience was a significant factor influencing the at-risk-of-PTSD group; for each unit increase in resilience, the odds ratio (OR) for being in the PTSD at-risk group declined by 0.91 (95% CI = 0.84–0.98).

A second model (Model II) was built, including the subfactors of resilience, social support, and PPN as the independent variables (χ^2^ = 81.97, *p* < 0.001) with an explanatory power of 61%. The factors influencing the at-risk-of-PTSD group included the subfactors of resilience (controllability and sociality) and social support (family). For each unit increase in resilience’s controllability, the OR for being in the at-risk-of-PTSD group fell by 0.60 (95% CI = 0.43–0.86), and for each unit increase in sociality, the OR declined by 0.68 (95% CI = 0.44–0.97). Additionally, for each unit increase in family support, the OR for being in the at-risk-of-PTSD group decreased by 0.47 (95% CI = 0.26–0.86) (Table 4).

## 4. Discussion

This study aimed to identify the effects of resilience, social support, and professional pride on PTSD among nurses during the COVID-19 pandemic. Resilience was a significant factor in the reduction of PTSD symptoms. Specifically, the subdimensions of controllability and sociality in resilience, as well as family support, were influential in reducing PTSD among nurses.

In this study, the average PTSD score was 16.21 ± 14.87, with 28.1% of participants categorized into the at-risk-of-PTSD group. Previous studies reported that in 2021, Korean nurses had an average PTSD score of 22.27, with approximately 35% falling into the at-risk group [19]. Efforts following the pandemic, such as infection education, management guidelines, reduced infection rates, and disease control, may have potentially alleviated trauma-induced stress among nurses. However, without statistical comparisons, we cannot definitively claim a reduction in such stress. Nevertheless, our findings indicated a higher proportion of at-risk-of-PTSD groups compared to those reported post-MERS and SARS outbreaks [23,24]. The rapid and prolonged COVID-19 pandemic, coupled with sustained anxiety, depression, and increased patient cases, likely contributed to this heightened risk among nurses [9,25]. Since the pandemic’s onset, nurses have continued to experience PTSD, highlighting its significance in public health. Given the evolving nature of PTSD post-trauma, longitudinal studies are essential.

Consistent with previous studies [7,18], the number of nurses with confirmed COVID-19 infections was higher among those who were at risk for PTSD than among those who were not. Nurses have higher infection rates due to the nature of their work, which involves frequent patient contact [4]. It is well known that nurses who provide direct care to patients are at greater risk of developing mental health issues [13]. Additionally, approximately 44% of nurses at risk for PTSD in this study were reassigned to specialized units caring only for COVID-19 patients. During the COVID-19 pandemic, nurses experienced sudden departmental reassignment owing to the establishment of isolation wards for patient management [26]. Stress and psychological burdens resulting from adapting to a new environment and a lack of time for recovery can lead to exhaustion, which may be a significant contributing factor to PTSD [27]. Building a sense and dynamic of teamwork under the leadership of nursing leaders and developing core competencies for emergency situations can mitigate the psychological challenges nurses encounter due to sudden reassignments [28]. 

Consistent with previous research, resilience is associated with decreased PTSD among nurses [13]. Factors such as good sleep quality, positive psychological resources, and high life satisfaction contribute to enhanced psychological resilience [29]. This can help reduce negative mental health outcomes, such as PTSD, and foster nurses’ internal growth. Simple training interventions can improve resilience. Moreover, online education, including mindfulness through three 20-minute videos, contributed to alleviating emotional distress, including PTSD, among healthcare workers during the COVID-19 pandemic [30]. Considering mental health intervention programs for nurses after the end of the COVID-19 pandemic, such programs can be swiftly implemented to promote resilience. Providing face-to-face interventions may not be feasible, as many Korean clinical nurses have rotating shifts [31]. Hence, there is a need to develop various online intervention programs to increase nurses’ participation. Organizational interventions aimed at enhancing resilience within healthcare institutions, along with individual nurses’ efforts, could be beneficial in effectively managing the nursing workforce.

Subcomponents of resilience, such as control and social support, were mitigating factors for PTSD among nurses. Control refers to the ability to regulate one’s behavior in challenging situations, including problem-solving, emotional regulation, and impulse control, whereas social support encompasses interpersonal relationships, communication, and empathy [20]. Nurses with a strong sense of personal control and psychological support derived from meaningful interactions in interpersonal relationships may find assistance in coping with PTSD.

During the COVID-19 pandemic, low social support was a significant risk factor for developing PTSD [13]. However, in the present study, social support was not a significant predictor of developing PTSD. This discrepancy could be attributed to the difference in the data collection periods between this study and previous ones. Past studies collected data during the COVID-19 pandemic, when the prevalence of PTSD in at-risk groups was considerably high, leading to significant results. By contrast, we collected data during the endemic period, resulting in a relatively lower prevalence of at-risk groups, which may have affected the statistical significance of our results. Further studies are required to validate the relationships between these variables. 

Family support, which is a subcomponent of social support, emerged as a factor that can mitigate the risk of developing PTSD. Additionally, the scores for support from family, friends, and significant others were significantly lower in the at-risk-of-PTSD group than in the normal group. Consistent with our findings, a study targeting nurses in California reported that high family support was associated with reduced PTSD [32]. Active family support during challenging times such as a pandemic can broadly help to mitigate negative psychological well-being, such as PTSD. Family support emerged as crucial during the pandemic due to restrictions on other forms of social support [33]. This highlights the need to explore further how family support specifically mitigates PTSD symptoms among nurses. Studies indicating a negative association between family support and insomnia [33], as well as resilience’s role in reducing nightmare distress and insomnia, suggest potential pathways through which family support can influence PTSD outcomes [34]. Therefore, future research could delve deeper into how different types of social support, particularly family support, interact with resilience to better understand their combined impact on PTSD among nurses. Moreover, discussing practical implications for healthcare settings, such as incorporating family support into mental health interventions, could strengthen the study’s relevance to clinical practice.

Professional pride among nurses was lower in the at-risk-of-PTSD group than in the control group. High professional self-identity among nurses can be associated with a sense of accomplishment stemming from the value of nursing care for COVID-19 patients, which may aid in overcoming PTSD [35,36]. Additionally, high professional pride among nurses can positively influence their coping abilities during stressful situations, such as disasters [16,36]. A strong professional identity as a nurse can positively impact personal growth after a crisis and may be linked to overcoming PTSD [36]. Although various studies have explored the correlation between PTSD and nurses’ satisfaction, sense of mission, and confidence in their profession, research measuring professional pride among nurses in Korea is lacking. As such, further research is needed to investigate the impact of professional pride among nurses, including those experiencing mental crises such as PTSD.

The limitations of this study are as follows: First, while this study utilized convenience sampling, which may not fully represent the entire nursing population in Korea in terms of gender and age, we aimed to include a diverse range of participants from multiple hospitals in a major city. Future studies employing random sampling methods could provide more representative insights into the broader nursing population demographics across Korea. Second, we targeted nurses working in tertiary general hospitals and hospitals with more than 300 beds. Subsequent studies should consider organizational culture, support budgets, and support systems based on hospitals of diverse sizes. Third, we did not take the participants’ emotional and mental states into account, which should be considered when interpreting PTSD levels. Future studies should limit the recruitment of participants with mental illnesses. Fourth, the period between the initial spread of COVID-19 and the gradual return to normalcy must be considered. As PTSD changes over time, longitudinal follow-up studies on its influencing factors are recommended. 

## 5. Conclusions

We aimed to investigate PTSD, resilience, social support, and professional pride among nurses during the COVID-19 pandemic, as well as identify factors influencing PTSD. The factors found to affect PTSD were subfactors of resilience, including control and social support, and family support, which is a subfactor of social support. To mitigate and prevent PTSD among nurses in the aftermath of a novel infectious disease outbreak, there is a need to develop and implement psychological intervention programs to enhance resilience. In addition, fostering positive relationships with family members could serve as a valuable source of psychological support for nurses experiencing PTSD. The factors identified in this study can inform the development and use of PTSD management programs for nurses. 

## Figures and Tables

**Table 1:** PTSD reported by nurses.

Categories	M ± SD, *n* (%)	Range
Hyperarousal	6.39 ± 4.91	0~24
Avoidance	4.24 ± 4.61	0~24
Intrusion	3.00 ± 3.42	0~20
Sleep disturbances, emotional numbness, and dissociation symptoms	2.58 ± 3.62	0~20
Total	16.21 ± 14.87	0~88
Normal (≤24)	8.41 ± 6.83, 87 (71.9%)	
High risk of developing PTSD (≥25)	36.15 ± 10.60, 34 (28.1%)	

**Table 2:** Risk of developing PTSD by general characteristics.

Variables	Categories (*n* = 121)			Normal (*n* = 87)		Risk of Developing PTSD (*n* = 34)		χ^2^	*p*
		*n*	%	*n*	%	*n*	%		
Gender	Male	9	7.4	5	5.75	4	11.76	1.29	0.257
	Female	112	92.6	82	94.25	30	88.24		
Age (years)	20–29	72	59.5	55	63.21	17	50	2.59	0.275
	30–39	38	31.4	26	29.89	12	35.29		
	≥40	11	9.1	6	6.9	5	14.71		
Education level	Associate’s degree	26	21.5	18	20.69	8	23.53	1.01	0.602
	Bachelor’s degree	88	72.7	65	74.71	23	67.65		
	Master’s degree or higher	7	5.8	4	4.6	3	8.82		
Work department type	COVID-19-dedicated ward	58	47.9	38	43.68	20	58.82	2.81	0.246
	General ward	14	11.6	12	13.79	2	5.88		
	ICU and ER	49	40.5	37	42.53	12	35.3		
Work schedules	Three-shift work	114	94.2	82	94.25	32	94.12	0.01	0.977
	Either day shift	7	5.8	5	5.75	2	5.88		
Position	Staff nurse	108	89.3	79	90.08	29	85.29	0.77	0.379
	Charge nurse and above	13	10.7	8	9.2	5	14.71		
Clinical experience (years)	5>	60	49.6	47	54.02	13	38.24	4.97	0.420
	5–10	32	26.4	21	24.14	11	32.35		
	11–15	14	11.6	10	11.49	4	11.76		
	>15	15	12.4	9	10.35	6	17.65		
Family type	Living with family	78	64.5	34	39.08	9	26.47	1.70	0.193
	Living with others	43	35.5	53	60.92	25	73.53		
Chronic diseases	Yes	10	8.3	7	8.05	3	8.82	0.02	0.889
	No	111	91.7	80	91.95	31	91.18		
Experience of being diagnosed with COVID-19 during the pandemic	Yes	100	82.6	67	77.01	33	97.06	6.85	0.009
	No	21	17.4	20	22.99	1	2.94		
Experience of being assigned to a dedicated department for COVID-19 patients	Yes	36	29.8	21	24.14	15	44.12	4.67	0.031
	No	85	70.2	66	75.86	19	55.88		
Average number of patients under one’s care during the pandemic	<10	30	24.8	19	21.84	11	32.35	1.45	0.229
	10≤	91	75.2	68	78.16	23	67.65		
Duration of caring for confirmed or quarantined COVID-19 patients (months)	<12	50	41.3	36	41.38	14	41.18	0.01	0.984
	12≤	71	58.7	51	58.62	20	58.82		
Changes in workload during the pandemic	Increased	102	84.3	72	82.76	30	88.24	0.55	0.457
	No change	19	15.7	15	17.24	4	11.76		
	Decreased	0	0	0	0	0	0		
Supply of personal protective equipment	Sufficient	103	85.1	75	86.2	28	82.35	0.42	0.812
	Insufficient	11	9.1	7	8.05	4	11.77		
	Unsure	7	5.8	5	5.75	2	5.88		
Experience of training in infection patient management	Yes	106	87.6	74	85.06	32	94.12	1.85	0.174
	No	15	12.4	13	14.94	2	5.88		
Presence of hospital guidelines for managing infectious diseases	Yes	110	90.9	80	91.95	30	88.24	0.41	0.522
	No	11	9.1	7	8.05	4	11.76		
Intention to care for patients with new infectious diseases	Yes	23	19.0	17	19.54	6	17.65	0.06	0.811
	No or unsure	98	81.0	70	80.46	28	82.35		

**Table 3:** PTSD risk by resilience, social support, and PPN.

Variables	Total	Normal	Risk of Developing PTSD	*t*	*p*
	M ± SD	M ± SD	M ± SD		
Resilience	85.45 ± 16.22	91.48 ± 14.42	70.00 ± 8.51	8.14	<0.001
Controllability	27.64 ± 5.88	29.20 ± 4.92	21.79 ± 3.67	8.70	<0.001
Positivity	28.30 ± 5.94	30.09 ± 5.90	23.71 ± 2.68	6.06	<0.001
Sociality	29.51 ± 5.60	31.47 ± 4.96	24.50 ± 3.77	7.40	<0.001
Social support	43.99 ± 9.77	47.34 ± 8.56	35.41 ± 7.14	7.21	<0.001
Family	14.65 ± 3.54	15.85 ± 3.15	11.59 ± 2.49	7.07	<0.001
Friends	14.62 ± 3.48	15.75 ± 3.02	11.74 ± 2.89	6.66	<0.001
Significant others	14.72 ± 3.25	15.75 ± 2.95	12.09 ± 2.42	6.43	<0.001
Nursing professional pride	82.65 ± 17.02	88.36 ± 14.60	68.06 ± 13.90	6.97	<0.001
Feeling of having a vocation	16.92 ± 4.43	18.33 ± 3.93	13.29 ± 3.52	6.52	<0.001
Role satisfaction	15.48 ± 4.63	16.32 ± 4.49	13.32 ± 4.33	3.33	<0.001
Role of being a problem-solver	20.87 ± 4.47	22.28 ± 4.07	17.26 ± 3.32	6.40	<0.001
Self-achievement	12.27 ± 3.00	13.16 ± 2.67	10.00 ± 2.63	5.88	<0.001
Willing to stay in one’s current position	17.12 ± 3.55	18.26 ± 3.09	14.18 ± 2.91	6.65	<0.001

**Table 4:** Factors influencing nurses’ risk of developing PTSD.

Variables	Categories	Model I		Model II	
		OR	95% CI	OR	95% CI
Experience of being diagnosed with COVID-19	Yes	5.51	0.60~50.36	3.09	0.26~36.75
	No (reference)				
Experience of being assigned to a dedicated department for COVID-19 patients	Yes	2.07	0.62~6.90	2.20	0.36~13.54
	No (reference)				
Resilience		0.91 *	0.84~0.98		
Social support		0.96	0.88~1.04		
Professional pride in nursing		0.98	0.93~1.03		
Resilience	Controllability			0.60 *	0.43~0.86
	Positivity			1.23	0.91~1.67
	Sociality			0.68 *	0.44~0.97
Social support	Family			0.47 *	0.26~0.86
	Friends			1.45	0.93~2.28
	Significant others			1.26	0.75~2.11
Professional pride in nursing	Feeling of having a vocation			0.71	0.46~1.08
	Role satisfaction			1.25	0.96~1.62
	Role of being a problem-solver			1.06	0.76~1.47
	Self-achievement			0.99	0.63~1.56
	Willing to stay in one’s current position			0.84	0.58~1.24

Model I: Omnibus tests of model: χ^2^ = 63.22 (*p* < 0.001), −2 log likelihood = 80.50, Cox and Snell’s R^2^ = 0.41, Nagelkerke’s R^2^ = 0.59, Hosmer–Lemeshow test: χ^2^ = 8.87 (*p* = 0.35). Model II: Omnibus tests of model: χ^2^ = 81.97 (*p* < 0.001), −2 log likelihood = 61.75, Cox and Snell’s R^2^ = 0.48, Nagelkerke’s R^2^ = 0.61, Hosmer–Lemeshow test: χ^2^ = 10.01 (*p* = 0.26). *: significant variables.

## Data Availability

The datasets analyzed during the current study are available from the corresponding author upon reasonable request due to privacy concerns.
